# A novel porcine bocavirus harbors a variant NP gene

**DOI:** 10.1186/s40064-015-1155-8

**Published:** 2015-07-24

**Authors:** Sung J Yoo, Sun Young Sunwoo, Seong Sik Ko, Sang H Je, Dong Uk Lee, Young S Lyoo

**Affiliations:** Department of Pathology, College of Veterinary Medicine, Konkuk University, 120 Neung-dong Street, Gwangjin-gu, Seoul, 143-701 South Korea; Department of Diagnostic Medicine/Pathobiology, College of Veterinary Medicine, Kansas State University, Manhattan, KS 66506 USA

**Keywords:** Porcine bocavirus, Phylogenetic incongruence, NP1 gene truncation, Recombination, Surveillance

## Abstract

**Background:**

Porcine bocavirus is classified within the genus *Bocaparvovirus*, family Parvoviridae. Unlike other parvoviruses, the members of genus *Bocaparvovirus* (bocaparvoviruses) encode an additional open reading frame (NP1). Many strains of PBoVs have been identified in domestic pigs and recognized as a potential emerging pathogen causing respiratory and gastrointestinal disease.

**Findings:**

A new strain of porcine bocavirus (PBoV) that harbored the shortest NP1 gene among all currently characterized PBoVs (provisionally named as ‘PBoV-KU14’) was detected in domestic pigs. Almost the complete genome sequence was obtained, approximately 4,630 nucleotides in lengths with putative NS1, NP1, and VP1/2 genes of 1,908, 600, 1,851 bp, respectively. Phylogenetic and comparative analysis was performed using protein and nucleotide sequences. It was revealed that PBoV-KU14 belongs to the genus *Bocaparvovirus* and species *Ungulate bocaparvovirus 4.* However, phylogenetic incongruence was observed among species classifications based on the NS1, NP1 and VP1/2 proteins, which indicates a probability of crossover recombination. Conserved protein domains unique for genus *Bocaparvovirus* in NP1, VP1 protein were also detected.

**Conclusion:**

NP1 gene truncation supposed to be caused by cross over recombination was detected in a new strain of PBoV (PBoV-KU14). Considering high rates of substitution and recombination in parvovirus, periodic surveillance study to monitor genomic variation and find new strainsof PBoVs seems to be needed.

The family *parvoviridae* is divided into two subfamilies according to the range of infecting host such as *parvovirinae* (infecting vertebrae) and *densovirinae* (infecting arthropods). The subfamily *Parvovirinae* is subdivided into eight genera, *Amdoparvovirus, Aveparvovirus, Bocaparvovirus, Dependoparvovirus, Erythroparvovirus, Protoparvovirus,* and *Tetraparvovirus*, respectively. Parvoviruses have a small linear single stranded DNA genome of 5 kb in length and a non-enveloped capsid with icosahedral symmetry (Tijssen et al. 2011). Unlike other parvoviruses, members of genus *Bocaparvovirus* (bocaparvoviruses) have an additional third open reading frame (ORF) named NP1, which is located between the first ORF coding nonstructural protein (NS1) and the second ORF that encodes structural proteins (VP1 and VP2) (Tijssen et al. 2011).

The first Porcine bocavirus (PBoV) known as a porcine boca-like virus was detected in the lymph node of Swedish pigs with post-weaning multi-systemic wasting syndrome (PMWS). Thereafter, several different strains of PBoV have been detected in respiratory tract secretions, fecal, serum, and urine samples of pigs from various countries (Lau et al. 2011; Zeng et al. 2011; Cheng et al. 2010; Blomstrom et al. 2009). According to 2013 ICTV species classification criterion, which stated that the identity of NS1 amino acid (aa) sequences should be under 85% in order to be demarcated as different species, PBoVs were classified into four species (*Ungulate bocaparvovirus 2*–*5*) (Cotmore et al. 2013).

In this study, we identified a novel porcine bocavirus (PBoV-KU14) in domestic pigs with respiratory problems, which has the shortest NP1 gene among all reported porcine bocaviruses. A phylogenetic and comparative analysis of PBoV-KU14 with other bocaparvoviruses indicated a possibility of crossover recombination. We also report conserved protein domains found in bocaparvoviruses.

One hundred and twenty-three serum samples from domestic pigs with respiratory problems or diarrhea in South Korea in 2012 were used to survey PBoV prevalence. Two degenerate primers (PBoV_F, PBoV_R), targeting the end of the NP1 gene and the beginning of the VP1/2 gene, were designed (Table 1) in order to classify viruses as either *Ungulate bocaparvovirus* (*UB*) *3/4* or *Ungulate bocaparvovirus* (*UB*) *2/5* species based on the size of DNA products. The expected PCR product size of *UB2/5* is ~900 bp and that of *UB 3/4* is ~720 bp, respectively.

**Table 1:** Ten sets of primers used to obtain nearly complete genome sequence of PBoV-KU14

Primer name	Sequence (5′–3′)
PBoV	F: KCACTTYAGATTTACTSMDTGT
	R: TTBAVDARYCWYTGCCAKTC
PBoV1	F: TGCTAAAAGGGCTAGAATG
	R: CCYGTBVMGTADATRTTBAG
PBoV2	F: GCGCAAATTCATCAGAACCA
	R: KHCCMMAYYKNGTTGGCATG
PBoV3	F: TGACCTAACCAGGTGGGGAA
	R: MVMWMWSMADWWDDSYKKBHT
PBoV4	F: GGDCCHGCCAGYACRGGNAA
	R: TGGTTGTGATGCCCACTTGT
PBoV5	F: GATCCGCTTTCTGATCTGCC
	R: ATTCTCACGGTTGTTCCTCCC
PBoV6	F: GTTTGGGGGATAGCGAGCAT
	R: CCTCCCGGCTGAGTTGTTTT
PBoV7	F: ATGTTTGGGGGATAGCGAGC
	R: AGGCAGATCAGAAAGCGGAT
PBoV8	F: GGAMRWAADDMTYRDWWWAAYAG
	R: TTTTGTGAGTCCGTCTCCTG
PBoV9	F: GTNTGGTGGGAAGAAGCRYTGA
	R: GCTGCCGTGTTCTTTGGATTT

The amplified DNA was run on a 1.2% agarose gel and the appropriate DNA band (720 or 900 bp, as mentioned above) was extracted using QIAquick Gel Extraction kit (QIAGEN) and subjected to sequence analysis (Macrogen, Korea). While twenty (16.3%) and fourteen samples (11.4%) showed a single positive band of 720 and 900 bp, respectively, three serum samples (2.4%) showed double bands (900 and 720 bp) indicating co-infection between the viruses of *UB3/4* and *UB2/5*.

Among 23 samples of 720 bp amplified PCR product, three sequences from each of three weaning pigs with respiratory problems in one pig farm had 72% maximum identity with prototype PBoV-H18 (HQ291308), and identity between these three sequences was 99–100%. One of the three samples was selected to obtain the full genome sequence and unknown 5′- and 3′-end sequences were obtained using a primer walking method (Kapoor et al. 2010) with nine sets of primers (Table 1). Each PCR amplicon for ten primer sets was sequenced in triplicate and assembled using Bioedit v7.2.5. A total of 4,630 bp without terminal sequences was obtained and submitted to GeneBank (accession number KJ622366). The remained two samples were used for the comparison of NP1 gene with that of PBoV-KU14 in aspect of length and nucleotide composition, which resulted in the same length and 99–100% identity.

The base composition of PBoV-KU14 was A (37.5%), C (19.0%), G (21.3%) and T (22.2%). Three open reading frames were identified using ORF finder (Rombel et al. 2002). The ORFs (NS1, NP1, VP1/2) of PBoV-KU14 comprised 1,908 bp (1–1,908 bp), 600 bp (2,136–2,735 bp), 1,851 bp (2,780–4,630 bp), respectively.

Phylogenetic analysis was performed using the molecular evolutionary genetics analysis (MEGA) v6 software in neighbor-joining (NJ) method mode with 1,000 bootstrap replicates. First of all, to identify relationships between PBoV-KU14 and other viruses belonging to the subfamily *Parvovirinae,* we performed phylogenetic analysis of the almost full-length nucleotide sequences, including from the start of the NS1 gene to the end of VP1/2. A total of 53 nearly complete genome sequences representing different parvovirus species of the subfamily *Parvovirinae* were used in this analysis. The results indicated that PBoV-KU14 was classified in the genus *Bocaparvovirus* (Figure 1). Additionally, phylogenetic trees based on the amino acid sequences of NS1, NP1, VP1/2 protein were also constructed to survey genetic distances between all known PBoVs (Figure 2).

**Figure 1:**
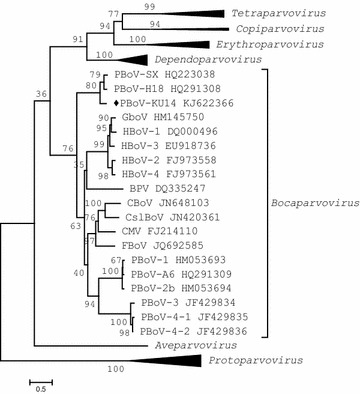
Phylogenetic relationships between viruses belonged to the family *Parvoviridae.* The phylogenetic tree was generated based on nearly complete genome sequences from members of subfamilies *Parvovirinae* (genera *Erythroparvovirus*, *Tetraparvovirus*, *Dependoparvovirus*, *Bocaparvovirus*, *Copiparvovirus*, *Aveparvovirus*, *Amdoparvovirus*, *Protoparvovirus*), using the neighbor-joining method. *Numbers* at the nodes indicate the values of 1,000 bootstrap analysis. PBoV-KU14 is marked by a *diamond*. *BPV* Bovine parvovirus, *CBoV* canine bocavirus, *CMV* *Canine* minute virus, *CslBoV* California sea lion bocavirus, *FBoV* feline bocavirus, *GboV* bocavirus gorilla, *HBoV* human bocavirus and *PBoV* porcine bocavirus.

**Figure 2:**
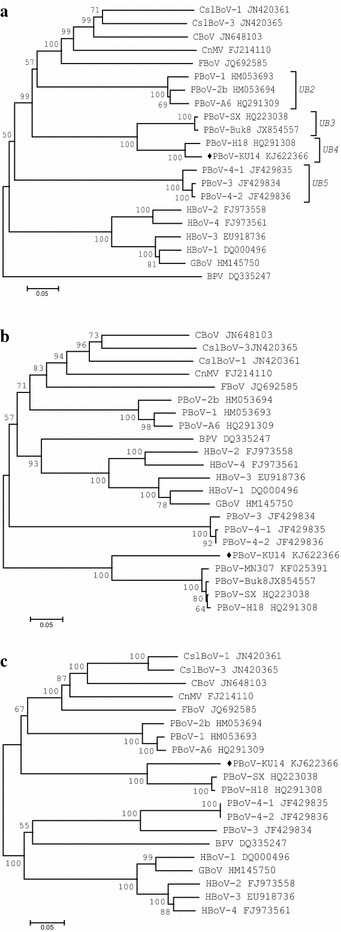
Phylogenetic relationships between viruses belonging to the genus *Bocaparvovirus.* The phylogenetic tree was generated based on protein sequences of NS1 (**a**), NP1 (**b**), VP1/2 (**c**), using the neighbor-joining method. *Numbers* at the nodes indicate the values of 1,000 bootstrap analysis. PBoV-KU14 is marked by a *black diamond.*

According to the ICTV criteria (Cotmore et al. 2013), PBoV-KU14 was identified as a new strain of *UB4* species (Figure 2a), showing amino acid identity of 94.9, 76.1% with PBoV-H18, SX, respectively. However, incongruent clustering was detected between the phylogenetic trees based on NS1, NP1 and VP1/2 proteins of PBoVs of *UB3* or *UB4*. The phylogenic incongruence was noticeable, especially in the classification of PBoV-KU14 (Figure 2), which highlights potential drawbacks with the current species classification system of ICTV. Amino acid identity of NP1 of PBoV-KU14 with those of PBoV-H18 and PBoV-SX was 58.1 and 58.9%, respectively; the identity of the VP1/2 proteins was 76.4 and 75.5%, respectively.

Comparative analysis of PBoV-KU14 with members of genus *Bocaparvovirus* was performed using tools for partial alignment (BLAST) (McGinnis and Madden 2004) and multiple alignment (ClustalW) (Thompson et al. 2002) with motif based sequence analysis tools (MEME suite) (Bailey et al. 2009). Partial alignment at the nucleotide level under *e*-value cutoff ≤10^−6^ revealed that nucleotides (nt) 1,909–2,049, 2,127–2,348, and 2,721–2,932 of PBoV-KU14 genome did not share any consensus sequence, which implies that these regions are not highly conserved. These domains include the 5′ terminal region of the NP1 (2,136–2,348 bp, 1–71 aa) and VP1 unique region (VP1u, 2,780–2,932 bp, 1–51aa). Otherwise, other regions of the PBoV-KU14 genome showed high identity with viruses of the *UB 3* or *4*.

Multiple alignment with protein motif analysis revealed that the region corresponding to 91-188 aa (2,406–2,699 bp) of the NP1 protein of PBoV-KU14 is highly conserved in the genus *Bocaparvovirus* and the VP1u protein of all bocaparvoviruses possesses a putative conserved C-terminal motif (Figure 3). Even though the protein functions of the conserved regions were not predictable due to insufficient online database of protein function, these findings provide additional insight into the general functions of the bocaparvovirus NP1 and VP1u protein.

**Figure 3:**
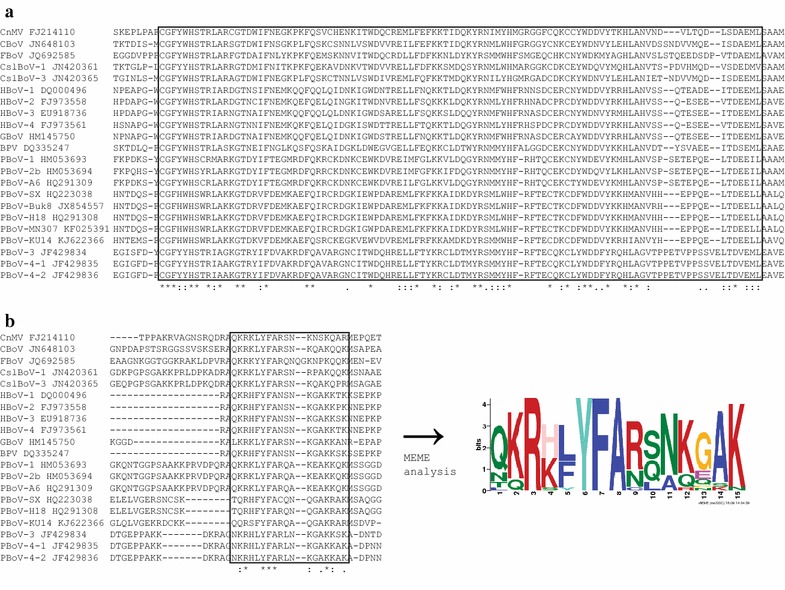
Conserved domain in NP1 protein (**a**) and expected protein motif in VP1u protein (**b**).

The length of each protein encoded by NS1, NP1, and VP1/2 ORFs are longest in viruses of the *UB2* strain and shortest in *UB3* and *4* viruses. While the length of NP1 protein is 218 aa in both *UB3* and *UB4*, the corresponding protein is 226 and 228–230 aa long in *UB5* and *UB2*, respectively (Table 2). Even though PBoV-KU14 belongs to the *UB4* strain, it expresses an NP1 protein that is 199 aa long. Through comparative analysis, the truncation could be attributed to the shorter 5′ terminal region of the NP1 gene (2,136–2,348 bp, 1–71 aa), which is an area of low conservation, than that of other members of the *UB3* and *4*. This shortening is due to two events. First, there has been a substitution at the start codon of the NP1 gene. In PBoV-KU14, the start codon of NP1 gene is replaced by ACA, with a consequent shift in translation initiation to a site 30 nt downstream of the original ATG. Second, a discontinuous deletion was detected in the 5′ terminal NP1 gene, which caused the NP1 protein of PBoV-KU14 to be 9 aa shorter (Figure 4). We presume that the 57 nt (i.e., 19 aa) truncation occurred on the basis of the two events mentioned above, and explain the changes to the length of NP1 protein.

**Table 2:** Comparison of the length of three encoded proteins (NS1, NP1 and VP1u/2) among different PBoV species

Species	Strain (accession number)^a^	The length of		
		NS1	NP1	VP1u/VP2
*UB2*	PBoV-1 (HM053693)	703aa	228aa	137/705aa
	PBoV-2b (HM053694)	703aa	230aa	137/709aa
	PBoV-A6 (HQ291309)	703aa	228aa	137/704aa
*UB3*	PBoV-SX (HQ223038)	636aa	218aa	68/623aa
	PBoV-Buk8 (JX854557)	637aa	218aa	NA
*UB4*	PBoV-H18 (HQ291308)	635aa	218aa	68/620aa
	*PBoV-KU14 (KJ622366)*	635aa	199aa	67/618aa
*UB5*	PBoV-3 (JF429834)	667aa	226aa	135/683aa
	PBoV-4-1 (JF429835)	667aa	226aa	135/683aa
	PBoV-4-2 (JF429836)	667aa	226aa	135/683aa

*NA* not available, *PBoV* porcine bocavirus and *UB* *Ungulate bocaparvovirus.*

^a^Strain found in this study was written in italics letter.

**Figure 4:**
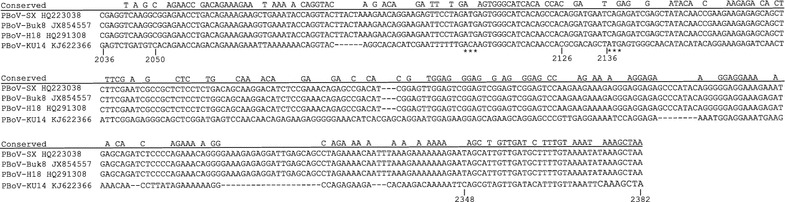
Comparison of PBoV-KU14 from 2,036 to 2,382 bp with the viruses of *Ungulate bocaparvovirus3,4* using Bioedit with ClustalW. Conserved residues are indicated above the alignments. The *numbers* indicated by *vertical bars* indicate the sequences of PBoV-KU14; the location where the suspected original start codon was changed to ACA and the start codon of NP1 gene start are marked by *asterisks.*

Viruses may achieve genetic diversity and adaptation through point mutation and recombination. Recombination leads to more rapid and massive viral evolution than point mutations, and results in either quasispecies or defective genomes. Previous reports of phylogenetic discord were attributed to recombination in parvoviruses (Yang et al. 2012; Ohshima and Mochizuki 2009; Kapoor et al. 2010; Tyumentsev et al. 2014) including the viruses of *UB5* (Xiao et al. 2013). Similar to other parvoviruses, through the results of phylogenetic and comparative analysis, we presume that the crossover recombination occurred before the region of the conserved NP1 gene during the speciation process. The truncation of the 5 terminal region of NP1 gene also seemed to be associated with recombination. Gene truncation is a phenomenon considered as host adaption that is found not only in pathogenic field strains of virus in in vitro cell culture systems (Pan et al. 2012; Steel et al. 2009) but also in naturally occurring viruses, such as the highly pathogenic porcine reproductive and respiratory syndrome virus (Yu et al. 2012). To determine whether this truncation is the result of viral adaptation to the host or is an independent event resulting from close contact between domestic pigs, periodic prevalence survey of PBoV-KU14 and studies of recombination occurring in PBoVs will be required. Additionally, because PBoVs have high intrinsic substitution rates (Babkin et al. 2013), and the chance that different PBoVs co-exist in one host is high, genetic change of PBoVs should be monitored carefully, and new strains should be subjected to persistent surveillance.
